# Validation of miRNAs as diagnostic and prognostic biomarkers, and possible therapeutic targets in medullary thyroid cancers

**DOI:** 10.3389/fendo.2023.1151583

**Published:** 2023-06-08

**Authors:** Alberto Mondin, Loris Bertazza, Susi Barollo, Maria Chiara Pedron, Jacopo Manso, Ilaria Piva, Daniela Basso, Isabella Merante Boschin, Maurizio Iacobone, Raffaele Pezzani, Caterina Mian, Simona Censi

**Affiliations:** ^1^ Endocrinology Unit, Department of Medicine (DIMED), University of Padua, Padua, Italy; ^2^ Laboratory Medicine, Department of Medicine (DIMED), University of Padua, Padua, Italy; ^3^ Department of Surgical, Oncological and Gastroenterological Sciences (DiSCOG), University of Padua, Padua, Italy; ^4^ Endocrine Surgery Unit, Department of Surgery, Oncology and Gastroenterology, University of Padua, Padua, Italy

**Keywords:** thyroid, medullary thyroid cancer, miR-21, PDCD4, miRNA silencing

## Abstract

**Introduction:**

Medullary thyroid cancer (MTC) is a rare type of neuroendocrine tumor that produces a hormone called calcitonin (CT). Thyroidectomy is the preferred treatment for MTC, as chemotherapy has been shown to have limited effectiveness. Targeted therapy approaches are currently being used for patients with advanced, metastatic MTC. Several studies have identified microRNAs, including miR-21, as playing a role in the development of MTC. Programmed cell death 4 (PDCD4) is a tumor suppressor gene that is an important target of miR-21. Our previous research has shown that high levels of miR-21 are associated with low PDCD4 nuclear scores and high CT levels. The aim of this study was to investigate the potential of this pathway as a novel therapeutic target for MTC.

**Methods:**

We used a specific process to silence miR-21 in two human MTC cell lines. We studied the effect of this anti-miRNA process alone and in combination with cabozantinib and vandetanib, two drugs used in targeted therapy for MTC. We analyzed the effect of miR-21 silencing on cell viability, PDCD4 and CT expression, phosphorylation pathways, cell migration, cell cycle, and apoptosis.

**Results:**

Silencing miR-21 alone resulted in a reduction of cell viability and an increase in PDCD4 levels at both mRNA and protein levels. It also led to a reduction in CT expression at both mRNA and secretion levels. When combined with cabozantinib and vandetanib, miR-21 silencing did not affect cell cycle or migration but was able to enhance apoptosis.

**Conclusion:**

Silencing miR-21, although not showing synergistic activity with TKIs (tyrosine kinase inhibitors), represents a potential alternative worth exploring as a therapeutic target for MTC.

## 1 Introduction

Medullary thyroid cancer (MTC) is a neuroendocrine neoplasm arising from thyroid parafollicular C-cells. It accounts for 2% of all thyroid malignancies, and 0.4-1.4% of all thyroid nodules (1). At diagnosis, patients with MTC often already have lymph node involvement and distant metastases (in around 35% and 13% of cases, respectively). The 10-year survival rates for patients with MTC stages I, II, III and IV are 100%, 93%, 71% and 21%, respectively (2). Primary treatment usually includes radical thyroidectomy with central neck lymph node dissection. Additional treatments are often needed, however, particularly in patients with distant metastases, but cytotoxic chemotherapy and radiotherapy have proved scarcely effective (3). MTC presents as a sporadic (75-80% of cases) (sMTC) or hereditary tumor (hMTC), as part of the multiple endocrine neoplasia 2 syndrome (MEN2) due to a germline REarranged during Transfection (RET) mutation.

On molecular characterization of sMTC using next-generation targeted sequencing (NGS), approximately 56% of patients carry a somatic RET mutation, 24% have a RAS gene mutation, and 2% have mutations involving other known genes, but around 18% test negative for any known genetic driver (4). An association has been established between the type of mutation involved in sMTC and a patient’s prognosis: it is worse in patients carrying a somatic RET mutation, Met918Thr (M918T), than in those with other RET mutations, and better in those with RAS gene mutations (5). It is therefore useful to assess patients’ gene mutation status for prognostic purposes, but also and more crucially to orient the choice of novel targeted treatments for advanced forms, such as the highly-selective RET inhibitors pralsetinib and selpercatinib, now approved for treating RET-mutated MTC (6), or crizotinib for use in the rare cases of ALK-rearranged MTC (7). Unfortunately, the genetic driver remains unknown in one in five patients with MTC, making it difficult to establish their prognosis and identify possible targeted therapies.

Managing advanced sMTC remains a challenge. Tyrosine kinase inhibitors (TKIs) (cabozantinib and vandetanib) have exhibited a variable degree of efficacy (8), but have not been shown to improve overall survival (9). Cabozantinib and vandetanib are nonetheless the drugs most commonly used to treat advanced MTC. Unfortunately, MTC often becomes resistant to TKIs, reducing their efficacy, with consequently worsening patient outcomes. These two drugs are also often poorly tolerated by patients, so the treatment frequently has to be discontinued or the dosage reduced due to adverse effects (2). The need for long-term treatment protocols because of the far from negligible adverse effects experienced by some patients, and the problem of drug resistance mechanisms that virtually all patients experience with time remain unresolved issues. There is therefore an urgent need for new, effective molecules or synergistic combinations to deal with these issues. Our incomplete knowledge of the molecular mechanisms behind all cases of sMTC prevents the design of a more effective treatment for advanced disease, particularly in cases with no known genetic drivers.

Micro-RNAs (miRNAs) are endogenous single-stranded noncoding RNAs that selectively bond to complementary 3’UTR mRNAs, influencing their cleavage and translation. Many studies have documented the involvement of miRNAs in the pathogenesis of cancer, including endocrine tumors (10, 11). A few studies, mainly by our group (12–16), have investigated the role of miRNAs in MTC. In recent years, the miR-21/PDCD4 pathway has been investigated as a possible therapeutic target in human tumors. Various anti-tumoral drugs take effect by inhibiting oncogenic miRNAs like miR-21, and thereby restoring the expression of tumor suppressor genes like PDCD4 (17, 18). *In vitro* and *in vivo* studies (19, 20), miR-21 overexpression has also been associated with chemoresistance in several human tumors - to cisplatin in tongue squamous cell carcinoma, for instance, and to gemcitabine in pancreatic cancer. No data on the possible role of miR-21 as a drug-actionable target in thyroid cancer are available as yet. In a previous translational study conducted by our group on a series of sporadic and hereditary MTCs, we demonstrated a significant inverse correlation between miR-21 overexpression and PDCD4 nuclear downregulation at tissue level. The miR-21/PDCD4 pathway was associated with patients’ clinicopathological variables and prognosis. High levels of miR-21 correlated with: high CT levels, lymph node metastases, advanced-stage MTC and persistent disease at the end of the follow-up. In parallel, nuclear PDCD4 down-regulation was associated with higher CT levels, advanced-stage MTC, and persistent disease at the end of the follow-up (16).

It has been demonstrated in studies *in vitro* and *in vivo* on many types of cancer that curcumin has an anticancer activity. It seems to take effect by reducing miR-21 expression (21, 22). Inspired by the therapeutic results obtained in other human cancers, we previously tested the anti-cancer activity of the curcumin analog EF24 in MTC cell lines (23). Our results showed that treating the MTC cell lines TT and MZ-CRC-1 with EF24 in addition to cabozantinib synergistically boosted the effect of the drug on: cell viability, phosphorylation of the MAPK pathway compounds at Western blot, CT secretion in the culture medium, and cell migration during wound healing experiments (23).

Based on these premises, the aim of our present study was to investigate the possible role of miR-21 as a novel therapeutic target in MTC. In particular, we used a specific anti-miRNA that targets miR-21 to suppress the expression of miR-21, examining the possible effects on two reference cancer cell lines for MTC: TT cells carrying a RET C634W mutation and MZ-CRC-1 cells carrying a RET M918T mutation. The effect of the anti-miRNA was studied alone and in combination with cabozantinib and vandetanib.

## 2 Materials and methods

### 2.1 Cell cultures and maintenance

The TT cell line (RRID: CVCL_1774) was obtained from American Type Culture Collection–LGC Standards S.r.l. (Milan, Italy). The MZ-CRC-1 cell line (RRID: CVCL_A656) was obtained from Dr. Alessandro Antonelli (Department of Clinical and Experimental Medicine, University of Pisa, Pisa, Italy). Both were cultured in RPMI 1640 (Gibco Life Technologies, Carlsbad, CA), supplemented with 10% fetal bovine serum, 2 mM L-glutamine (Sigma-Aldrich, Milan, Italy), and penicillin/streptomycin, as described elsewhere (24).

### 2.2 miRNA inhibitor

The anti-miR-21 (A-21) (Ambion, MH10206 – has-miR21-5p) and Negative Control #1 (Ambion, AM17010) were purchased from Life Technologies Italia. Cells were transfected with the anti-miR-21 molecule (a miR-21 inhibitor) and the Negative Control #1 for 6 hours using Lipofectamine RNAiMAX Transfection Reagent (Life Technologies Italia) according to the manufacturer’s protocols. The silencing mix was then removed and cells were maintained with RPMI 1640 supplemented with 8% FBS.

### 2.3 Drugs

EF24 was purchased from Sigma Aldrich, Italy. Cabozantinib (XL184) was purchased from Selleck Chemicals (Houston, TX). Vandetanib (ZD6474) was purchased from MERCK. The powders were dissolved in a 10 mM stock solution in dimethyl sulfoxide according to the manufacturer’s instructions, and stored at -80°C. During the experiments, every aliquot was thawed and used only once.

### 2.4 RNA extraction and cDNA synthesis

Total RNA was extracted from cells using the TRIzol reagent as the lysis buffer (Invitrogen, Carlsbad, CA, USA) according to the manufacturer’s protocol. RNA ex-tractions were performed using the Zymo DirectZol RNA Miniprep Kit (Zymo Re-search, cat. no. R2052) according to the manufacturer’s instructions. RNA was quantified by Nanodrop (Thermo-Fisher). cDNA synthesis was done with the TaqMan Advanced miRNA cDNA Synthesis Kit (Applied Biosystems), and the High-Capacity cDNA Reverse Transcription Kit (Applied Biosystems), depending on the type of assay to be performed.

### 2.5 Efficacy of anti-miR-21 silencing

The mirVana miRNA Inhibitors kit (ThermoFisher) was used to silence miR-21 on the two cell lines. This kit involves the use of specific miRNA inhibitors capable of targeting the miRNA of interest (miR-21 in our case). MiRNA inhibitors are single-stranded nucleic acids chemically modified to make them able to bond and inhibit specific endogenous miRNAs. These miRNA inhibitors are ready-to-use molecules that can be inserted in the cell using a liposomal transfection reagent or an electroporation protocol. We used a transfection agent (Lipofectamine® RNAi MAX Transfection Reagent, ThermoFisher Scientific), a formulation based on cationic lipids and designed specifically for short interfering (siRNA) transfection into cell lines. Silencing efficacy was tested using qRT-PCR for has-miR21-5p (miR-21; 5′-cgg tag cttatcagactgatgttg a-3′; ID: 477975_mir) on the StepOne real-time PCR system, with TaqMan advanced miRNA assays, according to the manufacturer’s instructions. Normalization was done by applying the hsa-miR-16-5p. All real-time reactions, including controls, were run in triplicate. Data were analyzed using the relative quantification method (2-ΔΔCT), as described elsewhere (25).

### 2.6 qRT-PCR for PDCD4 and CT

A real-time quantitative PCR (qRT-PCR) was performed in an ABI-PRISM 7900HT Sequence Detector (Applied Biosystems, Milan, Italy) using the relative quantification (2^-ΔΔCT^) method. The genes were analyzed using the following TaqMan assays: CALCITONIN (4331182; Hs01100741_m1); and PDCD4 (4331182; Hs00377253_m1), all from Applied Biosystems. Data were analyzed with the Sequence Detection Software rel. 2.4 (Applied Biosystems), adopting an automatically set baseline and a fluorescence threshold adjusted to measure quantification cycle (Ct) values. Using the 2^-ΔΔCT^ method, the data were presented as the fold-change in gene expression normalized by a reference gene and relative to a calibrator sample. As the reference gene in this study, we used β-actin (Hs99999903_m1), one of the most commonly used housekeeping genes.

### 2.7 Cell viability

Cells were plated on 96-well tissue-culture microtiter plates at a density of 4 × 10^4^ cells per well. The miR-21 inhibitor, and the respective controls were transfected to the cells for 6 hours, then the culture medium was removed, and cells were maintained with RPMI 1640 supplemented with 8% FBS. Next day, the cells were treated with the two drugs for 24 hours at various concentrations (cabozantinib at 1.4 µM for MZ-CRC-1 and 9.7 µM for TT; vandetanib at 10 µM for both cell lines). We measured the inhibitory effect on cell viability using 3-(4,5-dimethylthiazol-2-yl)-2,5-diphenyltetrazolium bromide (MTT) (Sigma-Aldrich). Then the culture media were removed and 100 µL DMSO was added to dissolve the formazan crystals, and absorbance was measured at 595 nm (Viktor 3 Perkin-Elmer). The signal intensity was then presented as a percentage relative to the control. Experiments were run in triplicate and repeated three times.

### 2.8 Western blot analysis

Cells were seeded at 4 × 10^5^ cells per well, then treated as described above for 4 and 24 hours to investigate the effects on PDCD4, pAkt and pERK expression: pAkt and pERK levels were measured after 4 hours due to the rapidity of the phosphorylation events, while PDCD4 was measured after 24 hours. Then samples were detached using trypsin 0.05%, washed with PBS and centrifuged at 8000 RCF for 5’ to collect the supernatant, which was immediately frozen in liquid nitrogen, and stored at -80°C. Immunoblot analysis was performed as described elsewhere (26). Briefly, proteins were separated with SDS/PAGE under reducing conditions in the presence of the S-S reducing agent dithiothreitol (DTT), then electroblotted onto nitrocellulose membranes and saturated in dry 5% non-fat milk. Membranes were incubated overnight with the primary antibodies (anti-phospho-Erk1/2 [RRID: AB_2315112], anti-PDCD4 [RRID: AB_2315112] and anti-GADPH [RRID: AB_370675], all purchased from Sigma-Aldrich, and all diluted 1:1000). Primary antibodies were detected with a secondary anti-mouse (1: 5000) and anti-rabbit (1: 10000) fluorescent antibody (IRDye 800CW, Li-Cor Biosciences, Milan, Italy). Membranes were scanned using LI-COR Odyssey Imaging Systems and the band intensity was quantified with LI-COR Empiria Studio® Software. The band intensity of each sample was normalized to its GAPDH signal in order to nullify any experimental differences in loading and to standardize the protein content loaded into the well. The normalized signal was then compared to the control. All experiments were performed in triplicate.

### 2.9 CT secretion

CT secretion was quantified in cells in the conditioned medium after treatment with the anti-miR-21 alone and in combination with the drugs. Briefly, cells were plated in 60 mm tissue culture dishes and treated for 24 hours. Then the conditioned medium was collected and the CT levels were quantified using LIAISON^®^ Calcitonin_II-Gen (DiaSorin Inc., Stillwater, MN, USA), an analytical method that employs a chemiluminescent immunoassay. CT concentrations were then normalized by cell number count for each well assayed. The experiments were performed in triplicate.

### 2.10 Wound healing

Cells were seeded on six-well plates at 4 × 10^5^ cells per well and treated for 48 hours with the drugs at one-half MTT IC50 doses. At the end of the treatment, the medium was removed and immediately replaced with complete culture medium, and a scratch wound was created using a pipette tip. Cell migration was then monitored for the next 72 hours with Leica DMI6000CS (Leica Microsystem, Wetzlar, Germany). Cells migrated into the wound surface, and the average distance of the migrating cells was measured with the Leica Application Suite (LAS-AF) 3.1.1. software (Leica Microsystems) and processed with ImageJ software (ImageJ 1.53e; Java 8 version [64-bit]) at 0 and 72 hours. The experiments were run in triplicate.

### 2.11 Cell cycle analysis

Cells were seeded on 12-well plates at a density of 1 × 10^6^ cells and treated with the drugs, alone or combined, for 48 hours using the mean IC50 doses ascertained by MTT, as described elsewhere (13). Then the cells were detached with trypsin, washed with PBS, and fixed with cold (4°C) 100% ethanol. The cells were washed with cold (4°C) PBS, treated with RNAse (final concentration: 1 mg/ml), resuspended in a solution of propidium iodide (PI, final concentration: 0.1 ug/ml in PBS), and incubated in the dark at 37°C for 1 hour. They were finally centrifuged, washed with PBS and read with the instrument. The cell-cycle analysis was done using multispectral imaging flow cytometry (ImageStreamX mark II; Amnis Corp, Seattle, WA, United States). The data were analyzed using the IDEAS 6.3 image analysis software (Amnis Corp). Images were compensated for fluorescent dye overlap by using single-stain controls. Image analysis was performed as follows: focused cells were distinguished by PI intensity on Channel 05 (Ch05) for identification of the G0/G1, S and G2/M phases. Subsequent image analysis on the G2/M phase enabled us to separate and quantify the mitotic cells.

### 2.12 Apoptosis

Apoptosis was assessed with the Caspase-Glo® 3/7 assay, a homogeneous, luminescent assay that measures the activity of caspase-3 and -7 (key caspases of apoptosis). Adding the Caspase-Glo reagent results in cell lysis followed by caspase cleavage of the substrate and generation of a luminescent signal produced by luciferase. The luminescence is proportional to the amount of active caspase. Cells were seeded in a 96-well white plate and treated as described before. On the day of the experiments, Caspase-Glo® 3/7 was prepared according to the manufacturer’s guidelines and added after 24 hours of treatment, following the manufacturer’s instructions. After shaking at 500 rpm for 30 seconds, the plate was incubated for 90 min at room temperature in the dark to stabilize the signal before measuring the luminescence with a luminometer (Viktor 3 Perkin-Elmer). Experiments were performed in triplicate and repeated three times.

### 2.13 Statistical analysis

All statistical analyses were performed using the MedCalc® Statistical Software version 20.027 (MedCalc Software Ltd, Ostend, Belgium; https://www.medcalc.org; 2022). The t-test was used to measure differences in miR-21 expression levels between the sample treated with anti-miR-21 and the Negative Control#1. The t-test was also used to measure differences in cell viability, PDCD4 and CT levels between the samples treated with the two drugs, alone and in combination with anti-miR-21. A p<0.05 was considered statistically significant.

## 3 Results

### 3.1 Effect of EF24 on miR-21 expression levels

**Figure 1:**
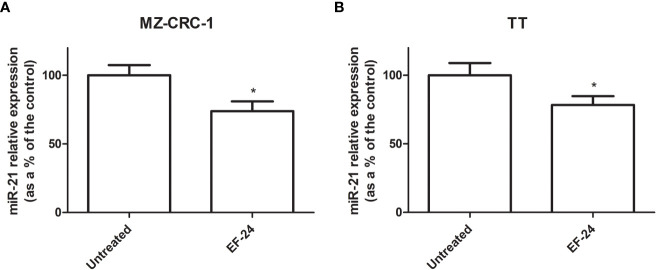
Bar graph showing miR-21 expression levels (as a percentage of the control) **(A)** MZ-CRC-1 cell line **(B)** TT cell line. *p < 0.05.

Treatment with EF24 reduced miR-21 expression by 26.2% in MZ-CRC-1 cell lines, and by 21.6% in TT cell lines, compared with the control (p<0.05, **Figure 1**).

### 3.2 miR-21 silencing efficacy

**Figure 2:**
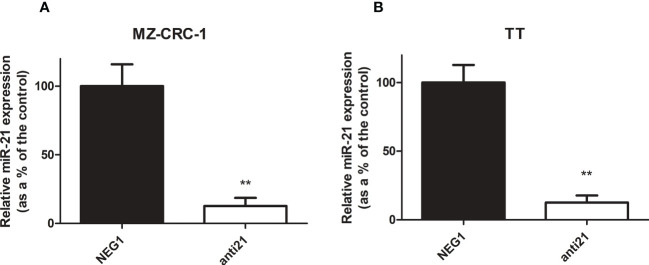
Relative miR-21 expression in MZ-CRC-1 and TT cell lines. **(A)** Relative miR-21 expression in MZ-CRC-1; (NEG1, Negative Control#1; anti21, anti-miR-21) **(B)** Relative miR-21 expression in TT; (NEG1, Negative Control#1; anti21, anti-miR-21). Experiments were performed in triplicate and repeated three times. Treatment vs. control: **p < 0.001.

We tested the efficacy of transfection in our *in vitro* models of MTC to obtain the starting point for our study. Transfection with anti-miR-21 using Lipofectamine RNAiMAX resulted in a downregulation of the miR-21 levels of 87.5% in the MZ-CRC-1 cell line (p<0.001), and 87.6% in the TT cell line (p<0.001, **Figure 2**).

### 3.3 Effects on mRNA expression levels

**Figure 3:**
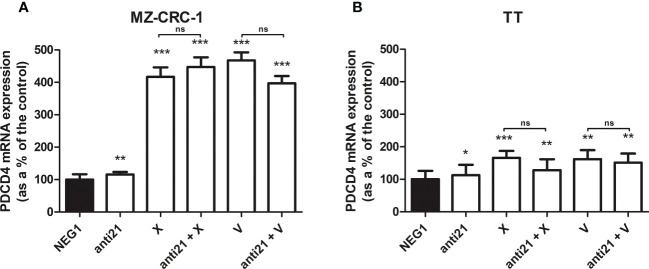
PDCD4 mRNA expression levels by quantitative real-time polymerase chain reaction (qRT-PCR) analysis. **(A)** MZ-CRC-1 cells; **(B)** TT cells; (NEG1, Negative Control#1; anti21, anti-miR-21; X, cabozantinib; anti21 + X, anti-miR-21 combined with cabozantinib; V, vandetanib; anti21 + V, anti-miR-21 combined with vandetanib). Experiments were performed in triplicate. *p < 0.05; **p < 0.01; ***p <0.001; ns, not significant. Treatment vs. control (unless stated otherwise).

**Figure 4:**
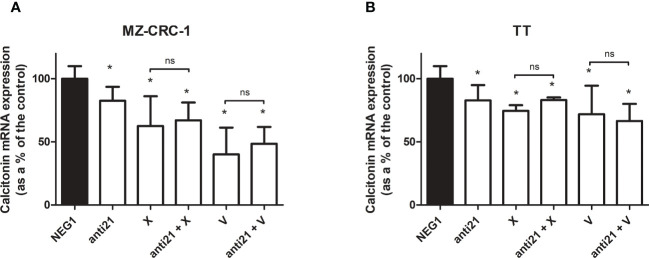
Calcitonin mRNA expression levels by quantitative real-time polymerase chain reaction (qRT-PCR) analysis. **(A)** MZ-CRC-1 cells; **(B)** TT cells; (NEG1, Negative Control#1; anti21, anti-miR-21; X, cabozantinib; anti21 + X, anti-miR-21 combined with cabozantinib 4; V, vandetanib; anti21 + V, anti-miR-21 combined with vandetanib). Experiments were performed in triplicate. *p < 0.05; ns, not significant. Treatment vs. control (unless stated otherwise).

We analyzed the effect of anti-miR-21 treatment on the expression levels of the onco-suppressor gene PDCD4 (**Figure 3**) and calcitonin (**Figure 4**). In the MZ-CRC-1 cell line treated with anti-miR-21, the expression of PDCD4 mRNA increased by 15.8% (p=0.0063) compared to the Negative Control#1; treatment with cabozantinib alone prompted the expression by 4.17-fold (p<0.001); and when the two treatments were combined, PDCD4 mRNA expression levels rose by 4,48-fold (p<0.001). No statistically significant difference emerged between the PDCD4 mRNA upregulating effects of cabozantinib alone and in combination. In the same cell line, vandetanib treatment prompted a 4.68-folds increase in mRNA PDCD4 levels (p<0.001) but combining it with anti-miR-21 did not increase its effect. Similarly in the TT cell line, treatment with anti-miR-21 alone increased PDCD4 mRNA expression by 12.9% (p=0.0196) compared with the control, and treatment with cabozantinib alone by 65.9% (p<0.001) but combining the two treatments did not add to the latter increase. Vandetanib likewise prompted a 61.9% upregulation in PDCD4 mRNA levels (p=0.002), but its combination with anti-miR-21 did not further raise its expression (**Figure 3**). Anti-miR-21 treatment resulted in a slight reduction in CT mRNA levels compared to the Negative Control#1: 17.5% in MZ-CRC-1 and 17.2% in TT (p<0.05). Cabozantinib reduced CT expression by 37.4% in MZ-CRC-1, and by 25.5% in TT (p<0.05). With vandetanib CT expression was reduced by 60.0% in MZ-CRC-1, and by 28.2% in TT (p<0.05). Associating anti-miR-21 with either of these two drugs did not result in a further reduction in CT mRNA levels (**Figure 4**).

### 3.4 Western blot analysis

**Figure 5:**
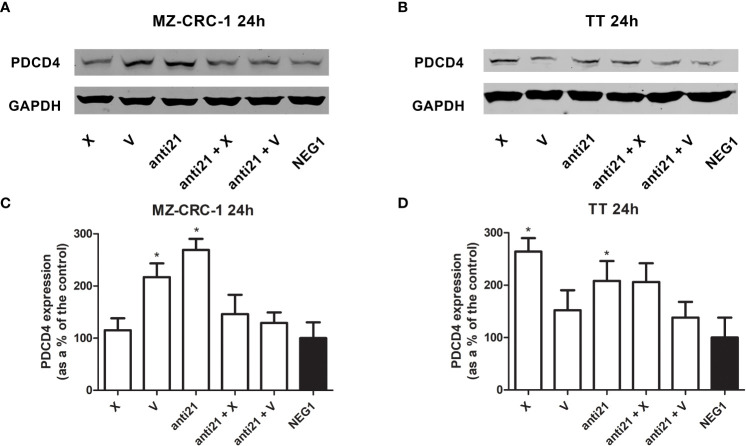
Representative Western blot analyses for PDCD4 in MZ-CRC-1 **(A)** and TT cells **(B)**; quantification of PDCD4 in MZ-CRC-1 cells; **(C)** quantification of PDCD4 in TT cells **(D)**; (NEG1, Negative Control#1; anti21, anti-miR-21; X, cabozantinib; anti21 + X, anti-miR-21 combined with cabozantinib; V, vandetanib; anti21 +V, anti-miR-21 combined with vandetanib). *p < 0.05. Experiments were performed in triplicate.

**Figure 6:**
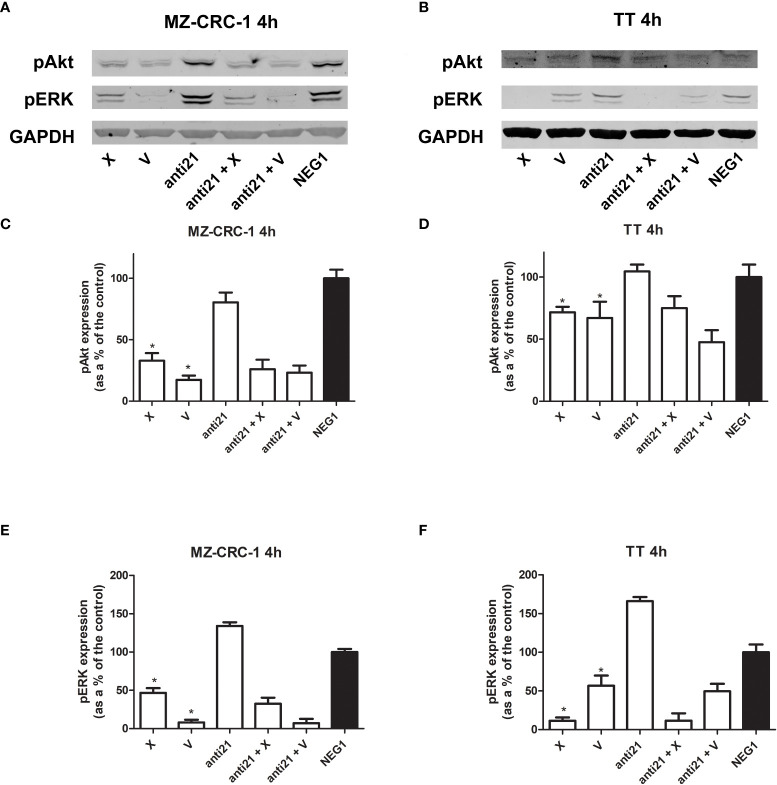
Representative Western blot analyses for MZ-CRC-1 **(A)** and TT cells **(B)**; pAkt and pERK were analyzed at 4 hours. Quantification of pAkt in MZ-CRC-1 cells **(C)**. Quantification of pAkt in TT cells **(D)**. Quantification of pERK in MZ-CRC-1 cells **(E)**. Quantification of pERK in TT cells **(F)**. (NEG1, Negative Control#1; anti21, anti-miR-21; X, cabozantinib; anti21 + X, anti-miR-21 combined with cabozantinib; V, vandetanib; anti21 +V, anti-miR-21 combined with vandetanib). *p < 0.05. Experiments were performed in duplicate.

Western blot was used to evaluate the effects of anti-miR-21 and the two drugs, alone or combined, on PDCD4, pAkt and pERK levels in the MZ-CRC-1 and TT cell lines. Inhibition of the phosphorylation signals was quantified, as shown in **Figures 5** and **6**, and each drug or combination was compared with its own Negative Control#1.

In the MZ-CRC-1 cell line, after 24 hours, anti-miR-21 raised the PDCD4 signal by 2.69-fold (p<0.05), compared to the Negative Control#1, and treatment with cabozantinib or vandetanib alone resulted in increases by 1.15-fold and 2.17-fold (p<0.05) respectively (**Figure 5**). However, combining anti-miR-21 with these drugs for 24 hours did not result in a further increase in PDCD4 expression. In the TT cell line, after 24 hours, anti-miR-21 increased the PDCD4 signal by 2.1-fold (p<0.05) compared with the Negative Control#1. Treatment with cabozantinib or vandetanib alone resulted in increases by 2.64-fold (p<0.05) and 1.52-fold, respectively compared with the Negative Control#1 (**Figure 5**). Adding anti-miR-21 did not modify the effect of either drug when it was used alone.

We analyzed the effects of anti-miR-21, alone and combined with the two drugs, on pAkt and pERK protein expression at 4 hours (**Figure 6**). Treatment with anti-miR-21 in the MZ-CRC-1 cell line prompted no change in the pAkt or pERK signals compared with the Negative Control#1. On the other hand, treatment with cabozantinib or vandetanib prompted a reduction of both pAkt and pERK levels: pAkt was reduced by 67.1% with cabozantinib (p<0.05), and by 82.6% with vandetanib (p<0.05); and pERK was reduced by 53.3% (p<0.05) with the former, and by 91.9% (p<0.05) with the latter. Adding anti-miR-21 boosted the effect of cabozantinib alone (the pAkt signal dropping further from 67.1% to 74.0%, and the pERK signal from 53.3% to 67.4%) but prompted no further decrease in pERK levels when combined with vandetanib.

In the TT cell line, treatment with anti-miR-21 for 4 hours again had no effect on pAkt but a slight increase in pERK levels, compared with the Negative Control#1. On the other hand, cabozantinib reduced the pAkt signal by 28.3% (p<0.05), and vandetanib lowered it by 32.9% (p<0.05), and the two drugs prompted a decrease in pERK levels of 88.6% (p<0.05) and 43.2% (p<0.05), respectively. Combining anti-miR-21 with cabozantinib made no difference, but its association with vandetanib boosted the effect observed with the drug alone, with a further reduction in Akt phosphorylation from 32.9% to 52.5%, and in ERK phosphorylation from 43.2% to 50.5%.

### 3.5 Effects of compounds on CT secretion of MTC cell lines

**Figure 7:**
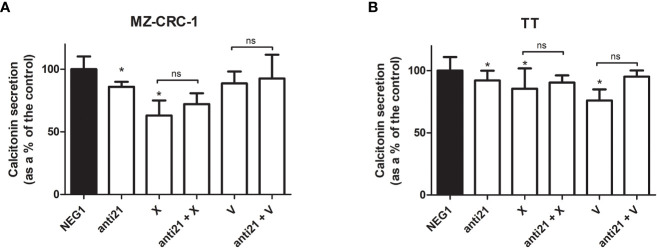
Calcitonin secretion levels. **(A)** MZ-CRC-1 cells; **(B)** TT cells; (NEG1, Negative Control#1; anti21, anti-miR-21; X, cabozantinib; anti21 + X, anti-miR-21 combined with cabozantinib; V, vandetanib; anti21 + V, anti-miR-21 combined with vandetanib). Experiments were performed in triplicate. *p < 0.05; ns, not significant. Treatment vs. control (unless stated otherwise).

We also investigated the effects of the drugs on the CT levels in the culture medium (**Figure 7**). Anti-miR-21 treatment lowered the concentration of CT compared with the Negative Control#1, by 14.1% in MZ-CRC-1, and by 8.0% in TT (p<0.05). Cabozantinib reduced CT levels by 36.9% in MZ-CRC-1, and by 14.4% in TT (p<0.05); and vandetanib reduced them by 11.3% in MZ-CRC-1 and by 23.9% in TT (p<0.05). In parallel with the qPCR results, combining anti-miR-21 with either drug did not result in a further reduction in CT concentrations.

### 3.6 Effects of compounds on MZ CRC 1 and TT migration

**Figure 8:**
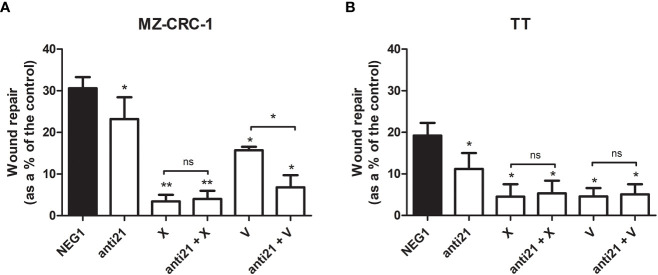
Effects on migration by wound healing analysis. **(A)** MZ-CRC-1 cells; **(B)** TT cells; (NEG1, Negative Control#1; anti21, anti-miR-21; X, cabozantinib; anti21 + X, anti-miR-21 combined with cabozantinib; V, vandetanib; anti21 + V, anti-miR-21 combined with vandetanib). *p < 0.05; **p < 0.01; ns, not significant. Experiments were performed in triplicate.

We performed an *in vitro* wound-healing assay to examine the effect of anti-miR-21, cabozantinib and vandetanib, alone and combined, on TT and MZ-CRC-1 cell migration, a core process in tumor growth (**Figure 8**).

Seventy-two hours after the end of the treatment, anti-miR-21 significantly inhibited the migration of both cell lines, which was reduced by 7.4% in MZ-CRC-1 (p<0.05), and by 8.0% in TT (p<0.05). In MZ CRC 1, wound repair reached 30.6% in the negative control, 23.2% with anti-miR-21 (p<0.05), 3.4% with cabozantinib (p<0.05), and 15.7% with vandetanib (p<0.05). Combining anti-miR 21 with cabozantinib did not further influence cell migration, but its combination with vandetanib led to a significant 6.8% further reduction in cell migration (p<0.05). In TT, wound repair reached 19.2% in the control, 11.2% (p<0.05) with anti-miR-21, 4.5% (p<0.05) with cabozantinib and 4.6% (p<0.05) with vandetanib. Adding anti-miR 21 to the cabozantinib or vandetanib treatment did not further influence cell migration.

### 3.7 Cell viability and apoptosis analysis

**Figure 9:**
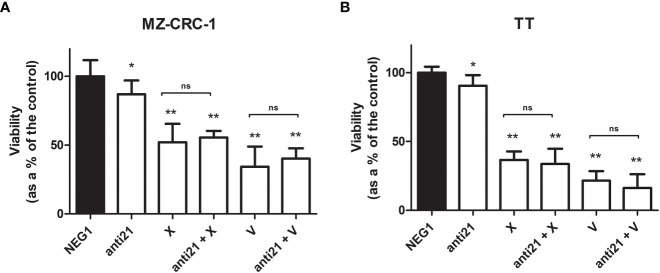
MTT assay for MZ-CRC-1 and TT cells treated for 48 hours. **(A)** MTT assay for MZ-CRC-1; (NEG1, Negative Control#1; anti21, anti-miR-21; X, XL184 1.4 µM; anti21 + X, anti-miR-21 combined with cabozantinib 1.4 µM; V, vandetanib: 10 µM; V + anti21, anti-miR-21 combined with vandetanib 10 µM). **(B)** MTT assay for TT; (NEG1, Negative Control#1; anti21, anti-miR-21; X, XL184 9.7 µM; anti21 + X, antimiR-21 combined with cabozantinib 9.7 µM; V, vandetanib: 10 µM; V + anti21, anti-miR-21 combined with vandetanib 10 µM). Experiments were performed in triplicate and repeated three times. *p < 0.05; **p<0.01; ns, not significant. Treatment vs. control (unless stated otherwise).

The effects of anti-miR-21, vandetanib and cabozantinib on MZ-CRC-1 and TT cell viability were examined *in vitro* at 48 hours, as described before. Using anti-miR-21 alone reduced cell viability by 13.1% in MZ-CRC-1 (p= 0.0066), and by 9.5% in TT (p= 0.0221). Using cabozantinib alone reduced cell viability by 48.0% in MZ-CRC-1 (p<0.001), and by 63.4% in TT (p<0.001). With vandetanib alone, the reduction in cell viability was 65.7% in MZ-CRC-1 (p<0.001) and 78.5% in TT (p<0.001). Combining anti-miR-21 with one of the other two drugs did not further influence cell viability in either MZ-CRC-1 or TT (**Figure 9**).

**Figure 10:**
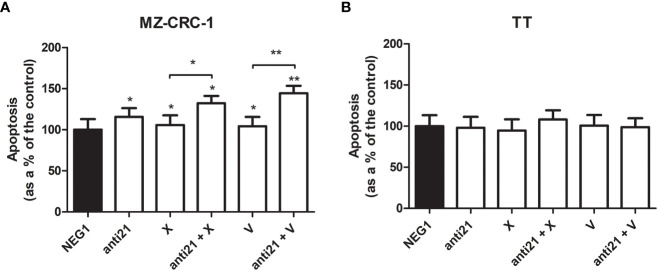
Effects on apoptosis on caspase assay. **(A)** MZ-CRC-1 cells; **(B)** TT cells; (NEG1, Negative Control#1; anti21, anti-miR-21; X, cabozantinib; anti21 + X, anti-miR-21 combined with cabozantinib; V, vandetanib; anti21 + V, anti-miR-21 combined with vandetanib). Experiments were performed in triplicate. *p < 0.05, **p < 0.01. Treatment vs. control (unless stated otherwise).

We examined the effect of anti-miR-21, cabozantinib and vandetanib, alone and combined, on MZ-CRC-1 and TT cell apoptosis, an important process that is downregulated in tumors (**Figure 10**).

After the treatment, apoptosis detected by chemiluminescence assay revealed a different behavior in the two cell lines. While no important effects were apparent in TT, the apoptosis rate in MZ-CRC-1 rose by 15.5% (versus the control) with anti-miR 21 treatment. Combining anti-miR 21 with either of the two drugs also increased the effects of the drugs alone, from a 5.8% boost with cabozantinib alone to 32.3% with the combination (p=0.001), and from 4.2% with vandetanib alone to 44.4% when it was combined with anti-miR 21 (p<0.001).

### 3.8 Cell cycle distribution

**Figure 11:**
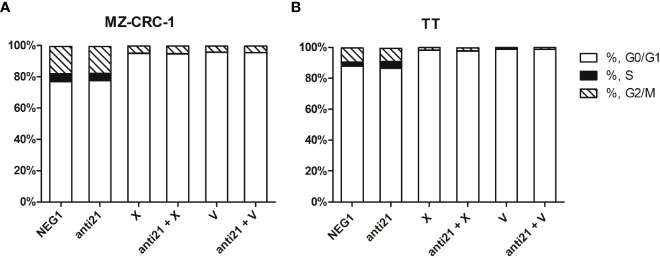
Effects on cell cycle. **(A)** MZ-CRC-1 cells; **(B)** TT cells; (NEG1, Negative Control#1; anti21, antimiR-21; X, Cabozantinib; anti21 + X, antimiR-21 combined with Cabozantinib; V, Vandetanib; anti21 + V, antimiR-21 combined with Vandetanib). Experiments were performed in triplicate and repeated three times.

**Figure 12:**
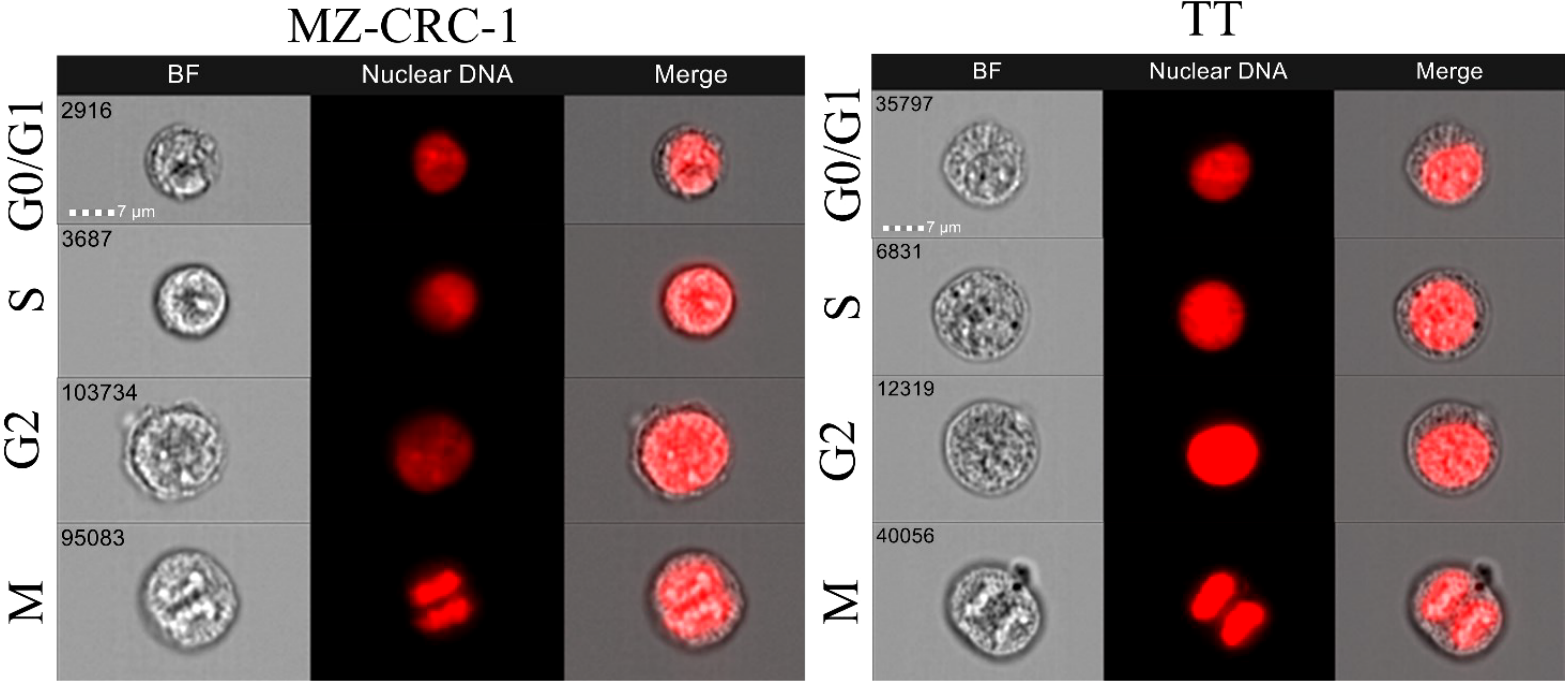
Representative images from the ImageStreamX analysis.

We used flow cytometry to analyze cell cycle distribution on the treated cells. (**Figures 11**, **12**). In MZ-CRC-1, the proportion of cells in the G0/G1 phase was 76.9%. The anti-miR-21 treatment was unable to increase this percentage (77.3%), whereas cabozantinib and vandetanib boosted it to 95.7% and 94.9%, respectively (p<0.001). Combining either drug with anti-miR-21 did not increase these percentages. In the TT cell line, the proportion of cells in the G0/G1 phase was 87.8%. Here again, treatment with anti-miR-21 made no difference (86.4%), whereas cabozantinib and vandetanib were able to boost this percentage to 98.0% and 98.7%, respectively (p<0.001). Adding anti-miR-21 to the drugs did not add to their effect alone.

## 4 Discussion

The focus of the present study was to investigate the possible role of miR-21 as a therapeutic target in MTC, alone or in combination with a TKI, through a series of experiments on MTC cell lines.

We wondered whether the promising results previously obtained in MTC cell lines using the curcumin analog EF24, which revealed a synergistic effect when added to cabozantinib, could also be achieved by directly silencing miR-21, a recognized curcumin target (23). For the first time in the literature, we demonstrated *in vitro* that treatment with EF24 effectively reduces miR-21 expression levels in MTC cells. We then targeted miR-21 directly with an anti-miR-21 treatment and analyzed its effects. To our knowledge, this is the first description of *in vitro* miR-21 silencing with an anti-miR-21 treatment in MTC cell cultures. The anti-miR-21 treatment was effective in reducing both MZ-CRC-1 and TT cell viability, albeit to a lesser extent than TKIs, and with a slightly more pronounced effect on the former cell line. Combining anti-miR-21 with either of the two drugs did not add to their effect. This result is in contrast with findings obtained using the curcumin analog EF24, which boosted the effect of TKIs on cell viability (23). We can thus assume that the synergistic effect of EF24 in combination with a TKI does not stem from the inhibition of its target miR-21.

We also demonstrated *in vitro*, for the first time, the association between miR-21 and PDCD4 in MTC, as suggested by our previous work at the tissue level (16). By silencing miR-21, we prompted an increase in the onco-suppressor PDCD4 levels in MTC cell lines, as seen directly by other research groups in breast cancer cell lines (27) and oral submucous fibrosis (28). Unexpectedly, we also demonstrated that cabozantinib and vandetanib can influence PDCD4 expression levels. Adding anti-miR-21 did not enhance the effect of the TKIs on PDCD4 protein levels at 24 hours. This means that both anti-miR-21 and TKIs can raise PDCD4 onco-suppressor protein levels but without any additive effect, so the mechanisms behind their effect are probably different. PDCD4 is a well-recognized miR-21 target, and the effect of anti-miR-21 is direct on its transcriptional machinery. The effect of TKIs is less specific as they have different TKI receptors acting on different pathways. Unlike the curcumin analog EF24 (23), anti-miR-21 had a low effect on Akt or ERK phosphorylation levels in our MTC cells. This would suggest that the effect of miR-21 on cell viability does not involve the key proteins acting along the MAPK pathway, so the effect of EF24 on these proteins may not involve the targeting of miR-21.

Treatment with TKIs was able to reduce CT mRNA expression and CT levels in the culture medium. The same effect was observed after treatment with anti-miR-21, although to a lesser extent. These results are intriguing because the CT gene is not a recognized direct target of miR-21, and TKIs are not known to influence CT production and secretion by C-cells. It should be noted that CT concentrations were normalized to the number of cells in the culture medium, indicating that the reduction in CT concentrations was due not to cell death, but to lower transcription and secretion. The outcome of the effect of anti-miR-21 and TKIs on CT secretion is an interference with its activity, although the underlying mechanism remains to be clarified. We can only speculate that it involves the regulation of other, still unknown intermediates.

Consistent with our findings on cell viability, treatment with anti-miR-21 reduced cell migration in our wound repair experiments, as demonstrated by Tao et al. (27). In the MZ-CRC-1 (but not the TT) cell line, adding anti-miR-21 to vandetanib also enhanced the drug’s effect on cell migration. We did not observe any effect of anti-miR-21 (alone or in combination with a TKI) on the apoptosis rate in TT cell lines. In MZ-CRC-1, on the other hand, anti-miR-21 treatment increased the apoptosis rate and considerably improved the effect of either TKI on cell apoptosis. Our findings on migration and apoptosis suggest that anti-miR-21 treatment is more effective in MZ-CRC-1 than in TT cell lines, although this difference could be partly explained by the former’s higher replication rate, which is associated with the very high risk of mutation carried by the MZ-CRC-1 cell line (M918T).

Anti-miR-21 functions by increasing cell apoptosis rather than regulating the cell cycle, as evidenced by the absence of any change in the proportion of cells in G0/G1 after anti-miR-21 treatment and the lack of any additive effect on the cell cycle when anti-miR-21 was combined with TKIs.

In our study, we employed a silencing system based on lipofectamine, which is a commonly used chemical vector for siRNA and anti-miRNA delivery that has been shown to be reliable (29). However, this method has limitations, one of which is that the silencing treatment is transient. This means that, as the cell divides over time, the original amount of anti-miRNA is gradually diluted, reducing its effectiveness. Although the slow cell proliferation rate of the MTC cell lines used can partly compensate for this limitation, the duration and complexity of the treatments may still be a weakness of our study.

In conclusion, our *in vitro* findings suggest that anti-miR-21 treatment could effectively reduce cell viability and cell migration, particularly in MTC cells harboring the M918T mutation, by increasing cell apoptosis. However, when anti-miR-21 was added to TKIs, it did not demonstrate any beneficial effect on any of these drug actions, except for boosting apoptosis and cell migration when added to vandetanib to treat the MZ-CRC-1 cell line. Anti-miR-21 treatment alone had an impact on PDCD4 protein levels and CT secretion, but combining it with TKIs did not affect the latter’s effect on these targets. Additionally, anti-miR-21 had no influence on the phosphorylation status of key proteins along the MAPK pathway or on the cell cycle in MTC cell lines. Our preliminary data confirm that the action of anti-miR-21 is very specific. The potential use of anti-miRNA as a therapeutic approach in human cancers is still a relatively new and unexplored area of research, particularly in thyroid tumors. This is due in part to unresolved issues with delivery strategies for RNA-based therapeutics in human cancers and the possibility of off-target effects resulting from the complexity of micro-RNA gene interactions (30). We speculate that, pending further studies, anti-miR-21 could be useful in the treatment of MTC in combination with drugs other than TKIs to reduce their effective doses and the resulting burden of side effects.

## Data availability statement

The original contributions presented in the study are included in the article/supplementary material. Further inquiries can be directed to the corresponding author.

## Author contributions

Conceptualization, AM, LB, SB, and CM; methodology, LB and SB; formal analysis, AM, LB, and MP; investigation, AM, LB, and MP; resources, DB and CM; data curation AM and LB; writing—original draft preparation, AM, LB, SB, and SC; writing—review and editing, MP, JM, IP, RP, IM, MI, CM, and SC; visualization CM and SC; supervision, SB and CM; project administration, CM and SC. All authors contributed to the article and approved the submitted version.
